# The pie‐crusting technique in knee surgery: Indications, technique, complications and outcomes

**DOI:** 10.1002/jeo2.70783

**Published:** 2026-05-26

**Authors:** Yavuz Şahbat, Serhat Akçaalan, Aybars Güzel, Kaya Turan, Safa Gürsoy

**Affiliations:** ^1^ Department of Orthopedics and Traumatology, Bahcesehir Liv Hospital Istinye University İstanbul Türkiye; ^2^ Orthopedics and Traumatology Department Ankara City Hospital Ankara Türkiye; ^3^ Department of Orthopaedics and Traumatology Faculty of Medicine Acibadem Mehmet Ali Aydinlar University Istanbul Türkiye

**Keywords:** knee surgery, medial collateral ligament (MCL), meniscus, osteotomy, pie‐crusting

## Abstract

Pie‐crusting represents a simple and widely utilised technique for controlled medial soft‐tissue release across various knee procedures. When applied in a gradual and indication‐driven manner, it can improve visualisation, facilitate instrumentation and reduce the risk of iatrogenic cartilage injury without causing clinically significant instability in most cases. However, the role of pie‐crusting differs among surgical contexts, including meniscal repair, periarticular osteotomy and knee arthroplasty, and should be tailored to procedure‐specific goals rather than applied routinely.

**Level of Evidence:** Level V, expert opinion.

**Abbreviations**
ACLR: anterior cruciate ligament reconstruction
dMCL: deep medial collateral ligament
MCL: medial collateral ligament
MOWHTO: medial opening‐wedge high tibial osteotomy
PC: pie‐crusting
POL: posterior oblique ligament
PROs: patient‐reported outcomes
sMCL: superficial medial collateral ligament
TKA: total knee arthroplasty

## INTRODUCTION

Medial collateral ligament (MCL) tension plays a critical role in both open and arthroscopic knee procedures, particularly with respect to visualisation of the medial tibiofemoral compartment and the achievement of appropriate soft tissue balance. In knees with a tight medial compartment, limited joint space may compromise safe instrumentation, increase the risk of iatrogenic cartilage injury, and restrict access to the posterior horn or posterior root of the medial meniscus. Similarly, in reconstructive and arthroplasty procedures, controlled modulation of medial soft tissue tension may be required to restore coronal alignment and achieve balanced knee kinematics. Various techniques have been described to address medial tightness, including subperiosteal release, capsular release and percutaneous ligamentous modulation. Among these, the pie‐crusting (PC) technique—characterised by controlled, incremental percutaneous puncturing of the MCL—has gained widespread acceptance due to its technical simplicity and reproducibility [8, 18, 58]. Despite its frequent application, important questions remain regarding the optimal indications, technical considerations, procedural context, and its true impact on stability and clinical outcomes.

The purpose of this narrative review is to comprehensively examine the role of controlled MCL release using the PC technique across different knee procedures. Specifically, indications, technical nuances and procedure‐specific applications in meniscal surgery, periarticular osteotomies, and knee arthroplasty, alignment strategies are analysed. In addition, potential complications, reported clinical outcomes and existing knowledge gaps are discussed in order to provide a structured and evidence‐informed perspective on this widely utilised yet insufficiently synthesised technique.

A narrative literature search was conducted using PubMed (MEDLINE) with the keywords ‘pie‐crusting’ AND ‘knee’. The search results were screened and evaluated by three experienced knee surgeons. Relevant studies were selected and categorised according to the thematic structure of the manuscript, including surgical technique, indications, complications and clinical outcomes. Some studies were included in more than one section when they addressed multiple aspects of the technique. This approach allowed for a comprehensive and structured synthesis of the available literature.

## WHEN SHOULD PC BE PERFORMED?

PC has been described as a safe and effective method for the controlled modulation of MCL tension, particularly in varus‐aligned knees where gradual correction of medial tightness may be required [9]. It should be considered in patients with a tight medial tibiofemoral compartment in whom adequate visualisation or instrumentation cannot be achieved despite standard manoeuvres. In varus knees, controlled medial release may additionally facilitate coronal plane correction by reducing excessive medial soft tissue tension [9].

Biomechanically, PC functions as a controlled tissue expansion technique achieved through multiple percutaneous perforations of the MCL. Previous studies have demonstrated that this approach may increase the medial joint space by up to 8.7 mm, thereby improving surgical access to the medial tibiofemoral compartment [19]. During diagnostic arthroscopy, the posterior aspect of the medial compartment remains one of the most difficult regions to visualise [18]. Limited working space in this narrow compartment may lead to technically demanding instrument manipulation and increase the risk of iatrogenic chondral injury.

Clinical practice patterns suggest that PC is used in approximately half of cases involving mid‐body medial meniscal lesions, whereas its utilisation approaches nearly universal application when posterior horn or posterior root access is required [18]. Standard valgus stress combined with external tibial rotation may not always provide sufficient joint space for safe visualisation of the posterior medial compartment. In most cases, a working space that allows comfortable passage of a probe is considered adequate. However, because baseline ligamentous laxity varies among individuals, the decision to perform PC should be made intraoperatively after assessment under extension and external rotation stress conditions [18] (Table 1 Cadaver).

**Table 1:** Summary of cadaveric studies evaluating the pie‐crusting technique.

	Country, publish year	Case	Finding
de Mont‐Marin et al.	France 2016	31 cadaver	Standardised medial collateral ligament (MCL) pie‐crusting technique produces a controlled and progressive increase in medial knee joint opening without causing ligament rupture, suggesting it is a safe and predictable method for ligament lengthening. (19‐gaugle)
Amundsen et al.	USA 2018	13 cadaver	MCL pie‐crusting progressively increases ligament length and reduces stiffness, but the exact effect varies between specimens and cannot be precisely predicted by a fixed number of punctures. needle size (16 v.s 18 gauge) did not significantly affect this
Seitz et al.	Germany 2018	6 cadaver	Releasing the MCL is mandatory to achieve the significant reduction in medial contact pressure and the desired lateralisation of the mechanical axis required for an effective medial open‐wedge high tibial osteotomy
Van Egmond et al.	Netherlans 2017	7 cadaver	No significant differences in mechanical stability, translations, rotations, or compressive strength between the relatively thin FlexitSystem and the firmer TomoFix locking plates, concluding that the FlexitSystem is a suitable biomechanical alternative for open wedge high tibial osteotomy
Pape et al.	Germany 2006	10 cadaver	The anterior fibres of the superficial MCL are crucial for maintaining valgus stability, leading researchers to recommend minimising ligament release during open‐wedge high tibial osteotomy to prevent potential post‐operative instability
Roussignol et al.	France 2015	10 cadaver	Performing a sequential pie‐crusting release of the superficial MCL at its distal tibial insertion—using the semitendinosus tendon as a reliable surgical landmark—provides a gradual and predictable opening of the medial compartment (up to 3.9 mm) while remaining anatomically safe for the saphenous nerve and medial saphenous vein
Rezaei et al.	USA 2024	7 cadaver	Grid‐assisted pie‐crusting technique using a 3D‐printed guide and an 18‐gauge needle allows for gradual and predictable lengthening of the medial and lateral collateral ligaments (averaging roughly 1 mm for the MCL) during total knee arthroplasty without sacrificing their structural integrity

## HOW SHOULD PC BE PERFORMED?

**Figure 1:**
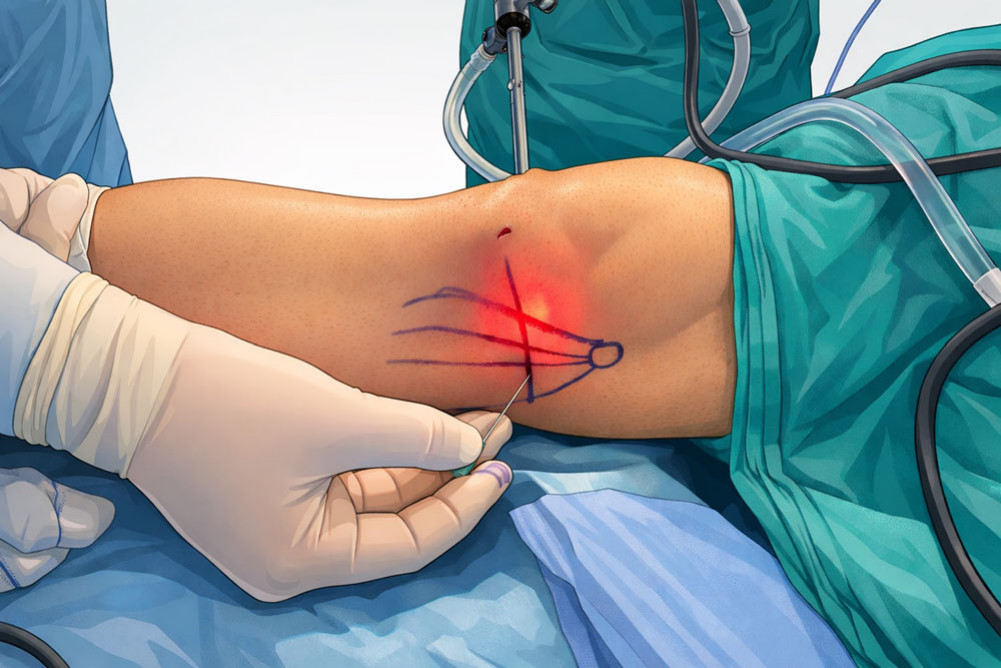
Illustration of the pie‐crusting technique performed on the medial collateral ligament using a 16‐gauge needle while the medial tibiofemoral compartment is visualised through the anterolateral portal under valgus stress.

The concept of controlled medial soft‐tissue release through PC evolved in the early 2000s. Agneskirchner and Lobenhoffer initially described a minimally invasive inside‐out approach aimed at releasing medial capsuloligamentous structures [1]. This technique was subsequently modified by Bosch et al. [11]. In the following decade, Bellemans and colleagues demonstrated that an outside‐in percutaneous needle technique could be effectively and safely applied, particularly in minimally invasive total knee arthroplasty, for gradual correction of medial tightness [9]. Since then, multiple variations of medial PC have been described; however, the most widely accepted and reproducible technique involves percutaneous release of the superficial MCL using a needle under controlled conditions (Figure 1).

Three principal target zones have been described for needle puncture: along the joint line, at the femoral origin of the MCL, or near the tibial insertion. Among these, incremental release performed close to the joint line is generally considered the most controlled and predictable method. A gradual release strategy remains the most accepted and safest approach. It has been reported that punctures performed approximately 5 mm proximal to the joint line, in sequential series, may provide stepwise correction of approximately 2–3° without inducing excessive instability [19]. Importantly, needle gauge does not appear to significantly influence the degree of medial laxity achieved. While Bellemans et al. utilised a 19‐gauge needle [8], Amundsen et al. reported no clear correlation between needle diameter (16‐ or 18‐gauge) and the magnitude of release [4, 9]. Instead, the number of punctures appears to be a more critical determinant of medial gap expansion [19].

Another technical consideration concerns which ligamentous fibres should be targeted. Both superficial and deep MCL fibres have been released in different studies. Arıcan et al. [6] demonstrated that releasing either superficial or combined superficial and deep MCL structures resulted in comparable clinical outcomes, similar protection against iatrogenic cartilage injury, and no significant difference in postoperative valgus laxity. Therefore, selective superficial MCL release remains a reasonable and commonly preferred strategy.

Both inside‐out and outside‐in techniques have been described. Atoun et al. [7] reported an inside‐out method using an 18‐gauge needle introduced through the medial portal to release the meniscocapsular structures from posterior to anterior. Javidan et al. [32] described the use of a banana blade introduced through the anterolateral portal to release the posterior third of the medial structures, while Leon et al. [40] utilised a hooked cautery device and Bert et al. [10] employed electrocautery and a microfracture awl. Although these techniques may provide adequate release, they may carry a higher risk of uncontrolled soft‐tissue damage when compared with controlled percutaneous needle puncturing. Limited reach of shorter needles or insufficient visualisation may also present technical challenges.

As experience accumulated, the trend shifted predominantly toward outside‐in percutaneous techniques due to their relative simplicity, reproducibility and perceived safety profile [12, 15, 21, 47, 52]. Moran et al. recommended performing the release under arthroscopic visualisation via the anteromedial portal, targeting a point approximately 1–2 cm posterior and 1–2 cm distal to the medial femoral epicondyle using an 18‐gauge needle [48]. However, no universal consensus exists regarding the exact distance from anatomic landmarks or the optimal number of punctures required.

Objective assessment of medial gap expansion has been explored in several studies. Roussignol et al. [56] arthroscopically measured medial compartment widening and demonstrated that selective release of the anterior, middle and posterior thirds of the MCL and posterior oblique ligament (POL) resulted in mean increases of approximately 1 mm, 2.3 mm and 3.9 mm, respectively. Intraoperative ultrasonography has also been proposed as an adjunct tool; Claret‐Garcia et al. [16] reported a mean medial compartment widening of 2.9 mm following PC. Alternatively, intraoperative or pre‐ and postoperative stress testing has been used to evaluate the adequacy of release [21, 26]. To ensure accuracy and safety, careful identification of anatomical landmarks is essential. Palpation of the medial femoral condyle, marking of surface landmarks and utilisation of arthroscopic transillumination through the skin may assist in precise needle placement while minimising the risk of neurovascular injury (Table 2).

**Table 2:** Technical pearls and pitfalls of medial collateral ligament (MCL) pie‐crusting.

Technical pearls	Technical pitfalls
Perform only when adequate visualisation cannot be achieved despite valgus stress and external tibial rotation.	Performing pie‐crusting without confirming true medial tightness.
Use gradual, incremental puncturing with frequent arthroscopic reassessment.	Excessive punctures without interval reassessment leading to over‐release.
Consider comfortable probe passage as a sufficient endpoint rather than maximal medial gapping.	Attempting to maximise medial opening instead of achieving functional working space.
Prefer selective superficial MCL release to preserve deeper stabilising structures.	Releasing deep MCL or posteromedial structures unnecessarily.
Favour outside‐in percutaneous technique under direct arthroscopic visualisation.	Using uncontrolled cutting devices (banana blade, cautery, awl) without sufficient experience.
Identify anatomical landmarks carefully; use palpation and transillumination to guide needle placement.	Inaccurate needle placement risking neurovascular irritation or haematoma.
Adjust release according to individual baseline ligamentous laxity.	Ignoring pre‐existing medial laxity and inducing postoperative valgus instability.
Focus on number of punctures rather than needle gauge.	Assuming larger needle diameter provides better control or predictable release.

## PC IN ARTHROSCOPIC MEDIAL MENISCAL SURGERY

### A stepwise, biomechanically guided release strategy

Arthroscopic surgery of the medial meniscus has evolved substantially over recent decades, driven by advances in instrumentation and an increasing emphasis on meniscal preservation. Despite these developments, access to the posterior horn and root of the medial meniscus remains technically demanding, particularly in knees with a tight medial compartment. Limited working space compromises visualisation, restricts instrument manoeuvrability, and increases the risk of iatrogenic chondral injury to the medial femoral condyle and tibial plateau during meniscal repair or meniscectomy [23, 63].

Controlled medial soft tissue release techniques were introduced to address these challenges by temporarily increasing medial joint space under valgus stress. Among these, percutaneous ‘PC’ of the MCL has gained widespread acceptance. Although initially described as a technical manoeuvre to facilitate exposure, accumulating biomechanical and clinical evidence suggests that PC should be conceptualised as a stepwise, anatomy‐ and function‐guided release strategy rather than a uniform technical intervention [33, 37].

This section synthesises current biomechanical, clinical and technical evidence supporting PC in medial meniscal surgery, with particular emphasis on a graduated POL–dMCL–sMCL release sequence designed to optimise visualisation while preserving medial knee stability.

### Biomechanical rationale for medial compartment release

The medial compartment of the knee is constrained by a complex capsuloligamentous system composed primarily of the superficial MCL (sMCL), deep MCL (dMCL) and POL. These structures act synergistically to resist valgus and rotational forces, with their relative contributions varying according to knee flexion angle [29, 39].

In the arthroscopic setting, valgus stress alone is often insufficient to overcome medial compartment tightness, particularly in patients with varus alignment, reduced ligamentous elasticity, or degenerative cartilage changes. Excessive valgus force may itself increase the risk of chondral injury without reliably improving visualisation [5].

PC involves controlled micro‐perforation of selected ligamentous fibres. By exploiting the viscoelastic properties of medial soft tissues, this technique allows temporary elongation without complete structural disruption. Cadaveric and in vivo studies using fluoroscopy and ultrasound demonstrate reproducible increases in medial joint space—typically ranging from approximately 1.9–5.7 mm—particularly when combined with controlled valgus stress [16, 37, 53].

Importantly, stress radiographic and comparative clinical studies consistently show that such controlled release does not result in clinically meaningful permanent valgus instability, with side‐to‐side differences generally within 0–1.1 mm or less than 1° [23, 33]. These findings support the concept that PC induces reversible ligament elongation rather than structural failure, thereby balancing surgical access with long‐term joint stability.

### Indications and patient selection

The primary indication for PC is a tight medial compartment that limits safe arthroscopic visualisation and instrumentation despite adequate valgus stress and portal optimisation. This scenario is particularly common during procedures involving the posterior horn, posterior root, or ramp region of the medial meniscus, where insufficient working space increases the risk of iatrogenic cartilage injury [63, 68].

Medial compartment tightness is more frequently encountered in middle‐aged or older patients, individuals with varus alignment, medial cartilage degeneration or reduced ligamentous elasticity [23].

PC should be regarded as a situational adjunct rather than a routine step, even in technically demanding posterior horn or root procedures. Contraindications include advanced medial compartment osteoarthritis, significant pre‐existing valgus instability, or severe malalignment in which the balance between benefit and risk may be unfavourable.

### Technical considerations: A stepwise POL–dMCL–sMCL strategy

**Figure 2:**
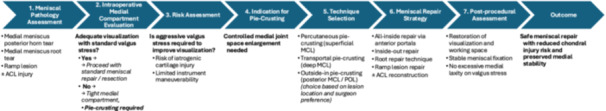
Pie‐crusting should be performed in a stepwise and controlled manner under arthroscopic visualisation. Technique selection may vary based on lesion location, knee alignment and surgeon experience. The algorithm reflects current evidence and expert consensus rather than a validated clinical pathway. (Level of Evidence: Level V).

Rather than a single uniform release, contemporary evidence supports a graduated, function‐guided approach targeting the structure primarily responsible for medial restraint at a given flexion angle. The overall decision‐making process is summarised in Figure 2. Integrating biomechanical principles into intraoperative assessment allows controlled, incremental compartment expansion while minimising the risk of over‐release.

#### POL: First‐line release

The POL functions as a key restraint to valgus and internal rotation, particularly near knee extension and in low degrees of flexion [17, 29, 39]. Limited posteromedial visualisation in near‐extension, despite acceptable opening in higher flexion angles, suggests POL‐dominant restraint.

Selective PC of the posteromedial capsule and POL, performed with the knee in 10–20° of flexion under controlled valgus stress, preferentially improves posterior compartment access. Biomechanical data demonstrate that isolated POL release significantly increases posteromedial joint space without inducing clinically relevant coronal plane instability when the sMCL remains intact [26].

Therefore, POL release represents the most conservative and biomechanically logical first step in tight medial compartments, particularly for posterior horn tears and ramp lesions.

#### Deep MCL (dMCL): Second‐line release

If medial compartment tightness persists in mid‐flexion (approximately 30–60°), the dMCL—comprising meniscofemoral and meniscotibial components—likely serves as the principal restraining structure. Arthroscopically, this manifests as persistent global medial narrowing despite adequate posteromedial release.

Targeted PC adjacent to the meniscocapsular junction produces a uniform increase in medial joint space, consistent with ultrasonographic and cadaveric findings [16, 53]. While isolated dMCL release may yield less expansion than sMCL release in severely tight knees, it provides a favourable safety profile when applied selectively.

#### Superficial MCL (sMCL): Final‐line release

The sMCL is the primary valgus stabiliser throughout the range of motion and should be reserved for cases in which adequate visualisation cannot be achieved following POL and dMCL release.

Percutaneous outside‐in sMCL PC produces the greatest increase in medial joint space. Although theoretical concerns exist regarding postoperative valgus laxity, systematic reviews and comparative studies consistently demonstrate that incremental, judicious sMCL release does not result in clinically meaningful residual instability [23, 33, 37].

When required, sMCL release should be performed conservatively and in small increments, guided by real‐time arthroscopic visualisation.

### Clinical outcomes and safety profile

Across retrospective cohorts, prospective observational studies and systematic reviews, PC has been associated with favourable clinical outcomes. Patient‐reported outcome (PRO) measures, including IKDC and Lysholm scores, demonstrate significant postoperative improvement, with preserved range of motion and return‐to‐activity timelines comparable to standard meniscal procedures [5, 20].

Importantly, adjunctive PC does not appear to compromise meniscal healing. In medial meniscal root repair, comparative studies report improved functional outcomes and, in some cohorts, lower retear rates when PC is employed, likely reflecting improved anatomic reduction and fixation under enhanced visualisation [28, 68].

Postoperative valgus instability remains the most frequently cited theoretical concern. However, objective stress radiographic assessments consistently demonstrate no clinically relevant residual laxity following controlled release. Reported complications are generally mild and transient, including localised medial pain, ecchymosis and temporary saphenous nerve irritation. Contemporary systematic reviews and comparative clinical studies consistently demonstrate favourable visualisation, improved functional outcomes and absence of clinically relevant valgus instability. A structured summary of these studies is provided in Table 3.

**Table 3:** Summary of systematic reviews and contemporary clinical studies evaluating pie‐crusting as an adjunct to arthroscopic meniscal surgery.

Author (Year)	Study type	Study Design/Level	Population	Meniscal pathology	Key findings	Clinical implications
Gaudiani et al.^23^	Systematic review	PRISMA; 4 studies, Level IV	192 patients	Isolated medial meniscal tears (repair or meniscectom)	Improved medial compartment visualisation; minimal residual joint space widening (0–1.1 mm); no clinically relevant valgus instability	Supports safety of percutaneous superficial medial collateral ligament (MCL) pie‐crusting during meniscal arthroscopy
Kotipalli et al.^37^	Systematic review	PRISMA; 15 studies, Level IV	1009 patients	Posterior horn tears, root tears, Ramp lesions, (± anterior cruciate ligament [ACL])	Mean intraoperative medial joint space increase 5.7 mm; no residual valgus laxity; lower reported chondral injury compared with non–pie‐crusting	Strongest current evidence supporting effectiveness and safety of pie‐crusting in meniscal surgery
Ercan et al. (2024)	Clinical comparative study	Prospective cohort–like design; Level II–III	Medial meniscal tears (K–L grade ≤2)	Partial meniscectomy	Pie‐crusting group showed significantly better IKDC, Lysholm and VAS scores at ≥2 years	Suggests functional benefit of pie‐crusting beyond visualisation alone
Herber et al.^28^	Clinical cohort study	Retrospective comparative; Level III	Medial meniscal root tears	Root repair with vs without pie‐crusting	Higher functional scores and lower retear rates in pie‐crusting group; no increase in valgus laxity	Indicates potential protective effect on anatomic root repair
Gabr and Robinson^22^	Clinical cohort study	Retrospective; Level III	Meniscal surgery ± ACL reconstruction	Medial meniscal tears	Pie‐crusting did not increase postoperative instability or complication rates	Confirms safety in combined procedures
Srisuwanporn et al.^62^	Clinical observational study	Prospective observational; Level II–III	Medial meniscal surgery	Medial meniscus tears	No clinically significant residual MCL laxity on stress radiographs	Addresses primary safety concern of pie‐crusting

### Discussion, limitations and future directions

The available evidence supports PC as a safe and effective adjunct in arthroscopic medial meniscal surgery when applied selectively. A key conceptual advance emerging from recent literature is the recognition that PC should be understood as a graduated, anatomy‐driven strategy, rather than a binary technical manoeuvre. As summarised in Table 3, the cumulative evidence—although largely Level II–IV—consistently supports the safety profile of incremental medial release.

The proposed POL–dMCL–sMCL sequence integrates biomechanical principles with intraoperative functional assessment, enabling surgeons to address compartment tightness precisely while minimising the risk of over‐release. To our knowledge, integrating this sequence into a structured, stepwise algorithm has not been clearly synthesised in previous narrative reviews.

Nevertheless, the current evidence base remains dominated by retrospective studies and heterogeneous patient populations, with randomised controlled trials lacking. Long‐term data evaluating the effects of controlled medial release on joint biomechanics, load distribution and osteoarthritic progression are limited [23, 37]. Future research should prioritise prospective comparative studies, standardised outcome reporting and biomechanical investigations quantifying load redistribution following sequential medial release.

### Conclusion

PC represents a valuable adjunct in arthroscopic medial meniscal surgery when standard valgus stress fails to provide adequate visualisation. Current evidence supports a stepwise, biomechanically guided POL–dMCL–sMCL release strategy that aligns surgical exposure with preservation of medial knee stability.

When applied judiciously in appropriately selected patients, PC enhances visualisation, facilitates precise meniscal repair and serves as a reproducible, tissue‐respecting adjunct within contemporary meniscus‐preserving surgery.

## PC IN PERIARTICULAR OSTEOTOMIES AROUND THE KNEE

Although PC is not routinely indicated during osteotomy procedures, controlled MCL modulation may be considered in selected cases in which excessive medial tightness persists despite adequate bony correction. Periarticular osteotomies around the knee, particularly medial opening‐wedge high tibial osteotomy (MOWHTO), are primarily bony realignment procedures; however, soft‐tissue balance remains a critical determinant of postoperative alignment and joint stability [50]. Unlike meniscal surgery or total knee arthroplasty—where MCL release is typically performed to improve visualisation or gap symmetry—the MCL is directly encountered within the surgical field during proximal tibial osteotomy and is almost inevitably manipulated during exposure.

Traditionally, access to the osteotomy site requires elevation of the anterior fibres of the superficial MCL in an L‐shaped fashion after mobilisation of the pes anserinus tendons [13]. Although this approach facilitates exposure, excessive release may theoretically predispose to medial laxity or unintended valgus shift. Alternative techniques have been described to optimise exposure while attempting to preserve stability. These include posterior retraction of the superficial MCL using a Hohmann retractor, creation of a secondary vertical window posterior to the superficial MCL to improve access to the posteromedial tibial cortex, and selective release beneath the plate using a scalpel when exposure remains limited [36]. More recently, a selective PC technique has been described in which MCL fibres located beneath the fixation plate (e.g., TomoFix) are carefully incised using a blunt‐tipped blade in a controlled manner [23]. In this context, partial or selective release may represent a more balanced strategy compared with complete detachment.

The biomechanical and clinical consequences of MCL release in osteotomy settings have been investigated with varying conclusions. Shim et al. reported that superficial MCL release did not result in valgus instability or valgus overcorrection at 1‐year follow‐up based on postoperative stress radiographs [61]. Similarly, Jun et al. found no significant differences in postoperative laxity or PROs between patients in whom the superficial MCL was transected versus preserved, suggesting that controlled transection may be technically simpler without compromising short‐term stability [35]. From a biomechanical perspective, Seitz et al. demonstrated that releasing the anterior and posterior fibres of the MCL significantly reduced medial tibiofemoral contact pressure—by approximately 25%—following osteotomy [59]. Van Egmond et al., in a cadaveric study, also reported that MCL release effectively decreased medial compartment contact pressures; however, concerns regarding over‐release and instability were raised, although the cadaveric data did not clearly substantiate clinically relevant instability [51, 55, 65]. Pape et al. further demonstrated in cadaveric stress testing that no significant difference existed between total excision and partial release of the superficial MCL, reinforcing the principle that only the minimal necessary release should be performed [51].

In MOWHTO, medial structures are frequently partially released during routine exposure. Therefore, additional PC should be reserved for cases in which intraoperative assessment reveals persistent asymmetric medial tension after wedge opening. Excessive release may compromise medial stability, particularly in patients requiring large correction angles. Importantly, unlike arthroscopic meniscal procedures—where the primary goal is improved visualisation—osteotomy‐related PC should be guided by alignment restoration and controlled ligament balancing rather than access alone. Given the limited high‐level evidence in this area, selective application, gradual release and careful intraoperative reassessment remain essential to avoid overcorrection or postoperative instability.

In cases of varus malalignment undergoing MOWHTO, particularly in patients with concomitant medial meniscus root tears, an alternative intraoperative strategy may be considered. Following initial arthroscopic evaluation of the meniscal root pathology, the procedure may be temporarily shifted to the osteotomy stage. During the exposure phase, an open release of the MCL is typically performed. After completing this step, the surgeon may return to the intra‐articular compartment to proceed with meniscal root repair. This sequence may provide sufficient visualisation of the medial compartment without the need for an additional PC technique. Therefore, in selected cases involving combined meniscal root repair and MOWHTO, open MCL release performed during osteotomy may eliminate the need for percutaneous ligament release while still allowing adequate surgical access. This approach represents the routine practice of the authors.

## PC IN KNEE ARTHROPLASTY

One of the recommended philosophies for total knee arthroplasty surgery is the mechanical alignment technique [44, 57]. This philosophy is based on the idea that alignment can be restored through soft tissue release and ligament balancing rather than bone cutting [31]. This balance can be achieved intraoperatively through various procedures: iliotibial band release, femoral recut, tibial recut, posterolateral capsule release, popliteus release, arcuate release and MCL PC [24, 46]. Achieving this balance is crucial for the clinical outcomes and survival of knee arthroplasty.

To restore balance in cases of varus deformity, the soft tissue sheath is gradually released from the proximal medial tibia. In the classic medial soft tissue release technique, the superficial and deep MCL are released from the proximal medial tibia using sharp subperiosteal dissection. If necessary, the semimembranosus tendon, posteromedial capsule, and pes anserinus tendons are also included in this release [30]. However, this method may cause over‐release in the flexion and extension gaps. Recently, it has been demonstrated that the PC technique for releasing medial soft tissue structures, which differs from this classic method, is safe and has no adverse effects on clinical outcomes, joint function and stability [45]. The PC technique has been frequently and easily applied in arthroscopic surgery and has reduced surgical complications [1]. This technique has been widely used in total knee arthroplasty, especially for releasing lateral knee structures, over the past 15 years and has recently begun to be used for releasing the MCL [49]. The aim of this technique is to provide relaxation of the medial structures while maintaining the structural integrity of the MCL [49]. In cases of severe medial contracture, the PC technique successfully provides relaxation of the medial structures and, thanks to its sensitivity, prevents excessive relaxation [27].

In TKA, the PC technique for MCL release can be applied in two different ways: needle puncturing and blade knife. In addition to these two techniques, some authors have recently indicated that the grid‐assisted pie‐crusting technique can also be used successfully [14, 54]. The PC technique performed using the needle puncturing method with 16‐gauge and 18‐gauge spinal needles has been shown to be effective and safe [27]. However, there are also authors who argue and state that the PC technique, especially when applied with a blade knife (11 blade), is not a reliable method [38]. In both techniques, 3–5 small incisions are made in the mid‐substance of the ligament with a needle or scalpel for release. It has been determined that the safe distance between these incisions should be 3–5 mm [4, 54]. It is recommended that the flexion and extension gaps be checked after every 5–10 needle punctures to prevent excessive release [8]. A distractor can be placed in the joint when using either a blade knife or needle puncturing. In the grid‐assisted technique, a grid that completely covers the MCL surface is designed. This grid is used as a reference for the PC procedure and contains numerous holes spaced 3 mm apart. The surgeon can safely apply the PC technique using these reference points [14]. Although previous studies have reported that the PC technique is successful for 2–4 mm of medial tightness, there are studies reporting successful lengthening up to 7.6 mm [46, 54].

Indications for the use of the PC technique in varus knees have been defined differently by different authors. In the study by Verdonk et al., PC is recommended for 6‐8 mm or less tightness in extension or flexion, while subperiosteal release is recommended for values above this [66]. In two separate studies, Bellemans recommended multiple needle puncture for tightness of 2–4 mm in extension and 2–6 mm in flexion [4, 8]. Ha and colleagues also recommended selective medial release technique using the PC for tightness of 1–10 mm in extension and subperiosteal release for any tightness in flexion [25].

In comparative studies, the classic release and PC techniques applied to varus knees were compared from different perspectives. While there was no difference between the two techniques in terms of PROs, it was shown that the use of constrained inserts was lower in patients who underwent the PC technique, and that it provided a safer release [3]. The effects of different release techniques on lower extremity alignment in varus knees were also evaluated, but no significant differences were found between the techniques [66]. However, some authors have reported that proximal tibial resection for medial varus showed better results in terms of both pressure balancing and PROs [64].

The use of robotic surgery in knee arthroplasty is increasing day by day. Robotic technology enables the personalisation of alignment in knee arthroplasty, adapting it to individual bone and natural ligament structures [60]. Some systems achieve this through preoperative 3D imaging, thereby facilitating implant positioning and sizing [60]. PC release can be easily used in an integrated manner with these robotic systems. Tension metres display real‐time ligament tension on the medial and lateral sides. When there is a mismatch greater than 2–3 mm between the medial and lateral sides, release can be successfully applied using the PC technique. Changes in the tension measuring devices after each perforation can be clearly seen, thus preventing over‐release.

## COMPLICATIONS

Although the PC technique was developed to minimise complications—particularly iatrogenic chondral injury associated with limited medial compartment visualisation—it is not devoid of potential risks [18]. Theoretical complications can be broadly categorised into two groups: (1) complications related to the needle entry site and (2) complications related to excessive ligamentous release.

### Needle entry‐related complications

Potential concerns include injury to the saphenous nerve or vein during percutaneous puncturing. However, available reports suggest that even with multiple punctures, clinically significant saphenous nerve injury has not been observed [30]. Similarly, the risk of saphenous vein injury appears to be minimal, with reported cases either absent or clinically insignificant [21, 42]. Minor subcutaneous haematoma may occur but typically resolves spontaneously within the first postoperative month. Transient medial knee pain in the early postoperative period has also been reported, generally resolving within 2 weeks without intervention [69].

Whether needle insertion results in measurable tissue injury or only subclinical microtrauma remains unclear. Although overt neurovascular complications appear uncommon, the extent of microscopic soft‐tissue disruption has not been fully characterised. Aldhilan et al. reported a case of heterotopic ossification (HO) following PC performed during anterior cruciate ligament reconstruction (ACLR) [2]. However, in the presented case, the location of HO was proximal to the documented puncture site, raising the possibility that the ossification may have been related to a concomitant subclinical femoral‐origin MCL injury rather than the PC procedure itself. Therefore, while HO represents a theoretical concern, a direct causal relationship with PC has not been definitively established.

### Ligamentous over‐release and instability

The primary concern among surgeons remains the risk of excessive MCL release, which may theoretically result in valgus instability. The superficial MCL serves as a primary restraint against valgus stress and contributes to external rotational stability, while the deep MCL provides restraint to valgus stress and internal rotation, particularly within the first 90° of flexion. Therefore, uncontrolled or excessive release may compromise medial stability.

Clinical data, however, suggest that controlled and incremental application of PC does not commonly result in symptomatic instability. Lons et al. [42] evaluated 40 patients who underwent PC during medial meniscal surgery and reported persistent joint line convergence angle (JLCA) widening at 6 weeks postoperatively without symptomatic instability. Similarly, Xu Han et al. [26] demonstrated a mean intraoperative medial widening of 2.2 mm, which persisted at 1 week postoperatively but returned to baseline by 6 months. These findings suggest that medial laxity may be transient and self‐limiting when release is performed in a controlled manner.

Importantly, the primary rationale for PC—reduction of iatrogenic chondral injury—has been consistently supported in the literature, with studies demonstrating a significant decrease in instrument‐related cartilage damage following controlled medial release [26, 41, 61]. When performed using an outside‐in percutaneous needle technique, PC appears to provide improved visualisation without translating into clinically relevant long‐term instability in short‐ to mid‐term follow‐up. Consequently, routine postoperative bracing solely for MCL protection has not been shown to provide additional benefit in most reported series [18, 34, 43].

### Instrument‐related considerations

Various instruments have been described for medial release, including 18‐gauge needles, banana blades, electrocautery hook devices and microfracture awls. Among these, percutaneous 16–18 gauge needles appear to offer the most controlled and reproducible release profile [18]. Cutting devices may achieve adequate release but carry a higher theoretical risk of uncontrolled tissue damage when not used carefully.

## CLINICAL OUTCOMES

Application of PC during ACLR to facilitate medial meniscal assessment and repair does not appear to adversely affect postoperative PROs or residual laxity [22]. Studies have shown no significant changes in tibial internal rotation or anterior tibial translation, and no detrimental effects on knee stability. Furthermore, PC has not been associated with a decrease in postoperative Tegner activity scores. By improving visualisation, the technique may facilitate surgical efficiency and allow more controlled instrumentation without introducing major complications. However, current literature does not demonstrate a consistent improvement in overall clinical outcomes or PROs attributable solely to PC.

Evidence appears more favourable in the context of medial meniscal surgery. Several studies report that PC performed during medial meniscal procedures is associated with significant improvements in Lysholm and IKDC scores, as well as higher rates of return to sports activities [67]. Regarding pain outcomes, approximately one‐third of patients may experience localised discomfort along the needle tract during the early postoperative period, typically within the first 2 weeks. Nevertheless, by 6 months postoperatively, pain levels in PC‐treated patients have been reported to be lower compared with non‐PC groups in several studies [15, 67, 69]. Importantly, there is currently no evidence in the literature indicating that residual laxity following PC necessitates secondary repair or reconstruction of the MCL [62].

## FUTURE DIRECTIONS

Despite the widespread use of PC across different knee procedures, several important knowledge gaps remain. Current evidence is largely based on retrospective studies and heterogeneous methodologies. Therefore, careful patient selection, incremental release and intraoperative reassessment remain essential to minimise complications. The potential effect of PC on medial meniscal extrusion, particularly in patients undergoing posterior root repair, has not been systematically investigated. Whether the degree of medial release influences postoperative extrusion or meniscal healing remains unknown. In addition, objective assessment of MCL integrity following PC has not been standardised. Advanced imaging modalities such as ultrasound‐based elastography may provide quantitative evaluation of ligament stiffness and healing; however, no prospective ultrasonographic or elastography studies currently exist in this context. Furthermore, the timeline and biological characteristics of spontaneous MCL recovery after controlled percutaneous release remain insufficiently defined. Clarifying the duration of transient laxity, the remodelling process of ligament fibres, and the long‐term biomechanical consequences of repeated or extensive release requires well‐designed prospective and imaging‐based investigations. Future prospective studies with standardised protocols are needed to better define indications, quantify biomechanical effects, and clarify long‐term clinical outcomes.

## SUMMARY

Limited visualisation of the medial tibiofemoral compartment is a common challenge during knee arthroscopy, particularly in patients with a tight medial compartment. Restricted joint space may hinder adequate assessment of the medial meniscus and articular cartilage and may increase the risk of iatrogenic cartilage injury during instrumentation. The PC technique, which involves controlled percutaneous puncturing of the MCL, has been described as a safe and effective method to temporarily increase medial joint space and improve arthroscopic visualisation.

When performed under valgus stress and with appropriate needle size most commonly a 16‐gauge needle the PC technique allows gradual and controlled release of the MCL fibres, resulting in widening of the medial tibiofemoral compartment. This increased working space facilitates safer instrumentation, improves visualisation of the posterior horn of the medial meniscus, and enables more precise meniscal repair or meniscectomy when indicated. Cadaveric and clinical studies have demonstrated that the technique can effectively increase medial joint space without causing clinically significant valgus instability or long‐term ligament insufficiency. Its role in knee around osteotomy procedures remains limited, and its contribution to robotic alignment strategies in TKA has yet to be clearly defined.

Furthermore, the PC technique may reduce the likelihood of iatrogenic cartilage damage that can occur when surgical instruments are introduced into a tight compartment. Current evidence suggests that, when applied with proper indications and technique, PC is associated with favourable clinical outcomes and a low complication rate. Therefore, the PC technique represents a valuable adjunct during knee arthroscopy in patients with a narrow medial compartment, providing improved visualisation and facilitating safer and more effective surgical management of medial compartment pathology.

## AUTHOR CONTRIBUTIONS

Yavuz Şahbat, Kaya Turan, Serhat Akçaalan and Safa Gursoy prepared the manuscript. Yavuz Şahbat and Aybars Güzel made the critical revision and searched related literature. Safa Gursoy and Yavuz Şahbat shaped the final structure of the manuscript. All authors in this study were fully involved in the study and preparation of the manuscript within has not been and will not be submitted for publication elsewhere.

## FUNDING INFORMATION

The authors have no funding to report.

## CONFLICT OF INTEREST STATEMENT

The authors declare no conflicts of interest.

## ETHICS STATEMENT

The authors have nothing to report.

## Data Availability

The data that support the findings of this study are available on reasonable request from the corresponding author.
